# Monitoring lung recruitability during recruitment maneuvers in children with respiratory failure on ECMO using electrical impedance tomography: case report

**DOI:** 10.3389/fped.2025.1545230

**Published:** 2025-02-19

**Authors:** Jan-Christoph Clausen, Annemarie Krauß, Antonia Schulz, Oliver Miera

**Affiliations:** ^1^ Department of Congenital Heart Disease—Pediatric Cardiology, Deutsches Herzzentrum der Charité (DHZC), Berlin, Germany; ^2^ Charité—Universitätsmedizin Berlin, Corporate Member of Freie Universität Berlin and Humboldt-Universität zu Berlin, Berlin, Germany; ^3^ Department of Congenital and Pediatric Heart Surgery, Deutsches Herzzentrum der Charité (DHZC), Berlin, Germany; ^4^ Berlin Institute of Health at Charité—Universitätsmedizin Berlin, Berlin, Germany; ^5^ DZHK (German Centre for Cardiovascular Research), Berlin, Germany

**Keywords:** lung recruitment, respiratory failure, children, ECMO, case report, EIT, HFOV

## Abstract

**Introduction:**

Currently, there are no clear guidelines regarding the use of recruitment maneuvers in pediatric patients with severe respiratory failure who are on extracorporeal membrane oxygenation (ECMO), nor is there consensus on how they should be performed.

**Methods:**

In this report, we describe pulmonary recruitment maneuvers performed in four children with respiratory failure on ECMO (three on VA-ECMO and one on VV-ECMO), all of whom were monitored using electrical impedance tomography (EIT) between March and December 2024.

**Results:**

Our findings demonstrate that EIT is a feasible tool for evaluating lung recruitability in children with severe respiratory failure on ECMO. Additionally, EIT aids in distinguishing between responders and non-responders to recruitment maneuvers.

## Introduction

Severe respiratory failure is associated with high morbidity and mortality especially when extracorporeal membrane oxygenation (ECMO) implantation is necessary (1, 2). To date, there are no clear guidelines on whether standardized recruitment maneuver (RM) should be applied in severe cases of respiratory failure requiring ECMO (3).

Some studies have evaluated the use of RM in adult and pediatric ARDS demonstrating that it can be feasible and effective in some patients (4–8). However, approximately 40% of patients did not respond to recruitment maneuvers, making it challenging to determine which patients would benefit from RM. Recent studies have shown that electrical impedance tomography (EIT) can be a valuable tool for optimizing mechanical ventilation in patients with ARDS on ECMO (9). Given the limited ability to monitor oxygenation changes during ECMO, EIT proves particularly valuable in this regard.

To the best of our knowledge, there is no existing data on the use of EIT to monitor lung recruitability in children with respiratory failure on ECMO.

We describe four patients in whom we assessed lung recruitability within the first 3 days on ECMO using electrical impedance tomography (EIT). The placement of the belts was consistent across all patients. The Enlight 2100 device (Timpel, São Paulo, Brazil) was used in all cases, along with neonatal or pediatric belts, depending on the patient’s thoracic circumference. The belts were positioned above the nipple line whenever possible, and their placement remained unchanged throughout the entire examination. Proximal flow sensors specific to the device were used to calculate changes in tidal volumes and lung compliance. Table 1 presents patient characteristics and changes in respiratory parameters.

**Table 1:** Patient characteristics are provided for all cases, including changes in respiratory parameters after recruitment maneuvers.

	Case 1	Case 2	Case 3	Case 4
Age	6 months	13 years	10 years	1 month
Weight (kg)	8	35	36	3.8
OI before ECMO implantation	46	17	38	73
OI after ECMO weaning	n.a.	11	10	9
Duration of ECMO run (h)	102	91	83	51
Type of ECMO	VA	VA	VA	VV
Effective RM	Yes	No	Yes	Yes
Changes within 1 h after RM				
Tidal volumes increased	Yes	No	Yes	Yes
Compliance improved	Yes	No	Yes	Yes
CO_2_ clearance improved	Yes	No	Yes	Yes
Dynamic lung strain increased	No	Yes	No	No

CO_2_, carbon dioxide; ECMO, extracorporeal membrane oxygenation; OI, oxygen index; RM, recruitment maneuver; VA, veno-arterial; VV, veno-venous.

Several studies have investigated the use of electrical impedance tomography (EIT) for assessing dynamic lung strain. Lung strain is defined as the difference in lung tissue deformation between two distinct pressure levels (e.g., varying levels of PEEP). While CT scans are considered the gold standard, EIT has demonstrated a reasonable agreement with CT scans when evaluating dynamic lung strain (10). The assessment of dynamic lung strain can be performed across different ventilatory modes, with high lung strain potentially contributing to ventilator-induced lung injury (11). Therefore, limiting dynamic lung strain during recruitment maneuvers is crucial. Most EIT devices are capable of displaying impedance values derived from EIT pixels at various time points within the respiratory cycle. These values, which reflect changes in lung impedance rather than those measured by flow sensors (as in conventional ventilators that calculate tidal volume or pressure), offer comparable insights. Notably, values based on impedance changes typically feature a small ‘z’ at the end of their abbreviation.

Care guidelines were followed for the manuscript preparation (12).

### Case 1

This was a 6-month-old female patient (8 kg) with a partial cavopulmonary connection (PCPC) due to an unbalanced atrioventricular septal defect (AVSD). She developed pediatric ARDS secondary to viral pneumonia and required urgent veno-arterial ECMO implantation during cardiopulmonary resuscitation due to severe respiratory failure, with the lowest arterial pH recorded at <6.7. The first chest x-ray after ECMO implantation showed diffuse lung infiltrates, and nearly no tidal volumes could be generated on mechanical ventilation, which was set in pressure-controlled mode with a PEEP of 10 cmH_2_O and driving pressures of 15 cmH_2_O.

A recruitment maneuver (RM) was performed using incremental and decremental PEEP titration, with EIT (P1-belt, Timpel, São Paulo, Brazil) used to monitor the intervention. We first increased PEEP by 2 cmH_2_O every 5 min up to a maximum of 24 cmH_2_O with fixed driving pressures of 10 cmH_2_O, followed by a gradual reduction back to the baseline PEEP of 10 cmH_2_O. Lung recruitability was assessed by observing changes in both EIT signals and conventional ventilator parameters, including tidal volumes and lung compliance.

During incremental PEEP titration, tidal volumes and lung compliance remained stable, which was interpreted as a sign of ongoing lung recruitment. Dynamic EIT images did not show any increase in dynamic lung strain in any region of interest. The patient remained hemodynamically stable throughout the procedure, and no adjustments to the ECMO settings were required.

**Figure 1:**
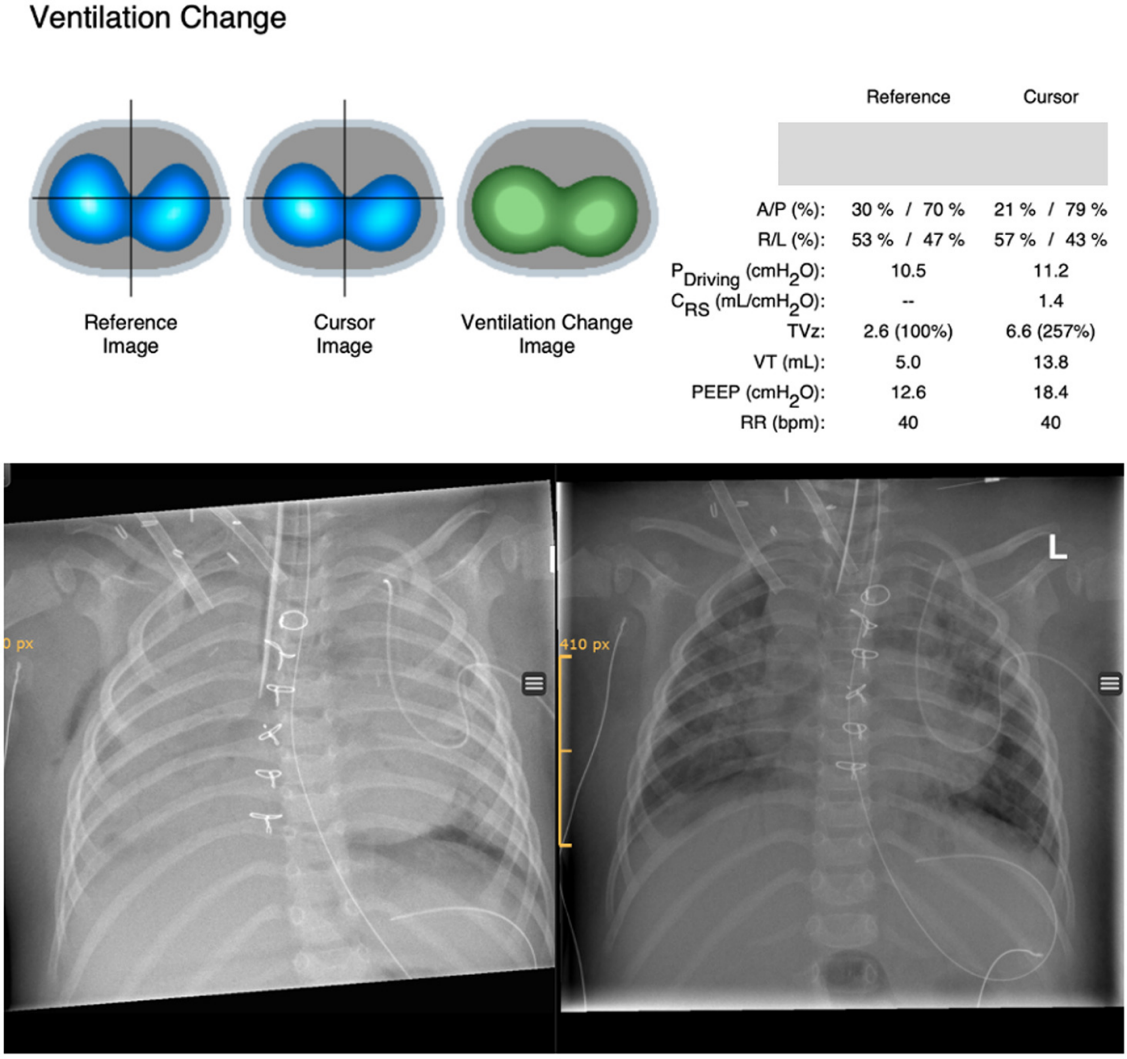
Ventilation changes derived from EIT are shown above. The reference image shows ventilation distribution before recruitment and the cursor image ventilation distribution after recruitment. The ventilation change image compares ventilation distribution before and after recruitment (reference image vs. cursor image) based on EIT pixels and displays changes in colors (green, gain in ventilation; red, loss in ventilation). Ventilator settings for paired time points and associated changes of different ventilatory and EIT parameters are shown on the right side comparing the same reference and cursor time points. Ventilation change after recruitment on ECMO with incremental/decremental PEEP titration up to 24 cmH_2_O showed a significant gain of ventilation (green) and improvement in tidal volumes. Chest x-ray before recruitment maneuver (left picture) and after recruitment (right picture). A/P, anterior/posterior; R/L, right/left; Pdriving, driving pressure; Crs, compliance respiratory system; TVz, tidal volume based on EIT signals; VT, tidal volume in milliliters.

With RM, tidal volumes (VT) increased from 0.5 ml/kg to 2.5 ml/kg during the decremental PEEP titration. EIT signals indicated a substantial improvement in ventilation (Figure 1). Post-intervention blood gas analysis showed improved gas exchange, and FiO_2_ and ECMO sweep gas flow were reduced by 30%. A chest x-ray performed immediately after the RM demonstrated a significant reduction in atelectasis.

However, due to significant neurological impairment following the cardiopulmonary resuscitation, confirmed by two independent pediatric neurologists, therapy was discontinued with parental consent, despite the improvements in pulmonary function.

### Case 2

This was a 13-year-old female patient with a body weight of 35 kg who received double lung transplantation due to drug-resistant pulmonary arterial hypertension. The day after transplantation, donor lungs showed decreased compliance with <0.5 ml/cmH_2_O/kg. The donor had a car accident, and lung contusion of the donor organ was suspected. We performed a recruitment maneuver as outlined before, starting from a baseline PEEP of 9 cmH_2_O and with a constant driving pressure of 10 cmH_2_O. We stopped at a maximum PEEP of 15 cmH_2_O because of the continuous worsening of lung compliance and dynamic EIT signals without recruitment effect over time. The patient remained hemodynamically stable throughout the procedure. Upon returning to the baseline PEEP of 9 cmH_2_O, EIT signals showed no improvement in ventilation. Decreasing PEEP to 6 cmH_2_O also yielded no benefit.

**Figure 2:**
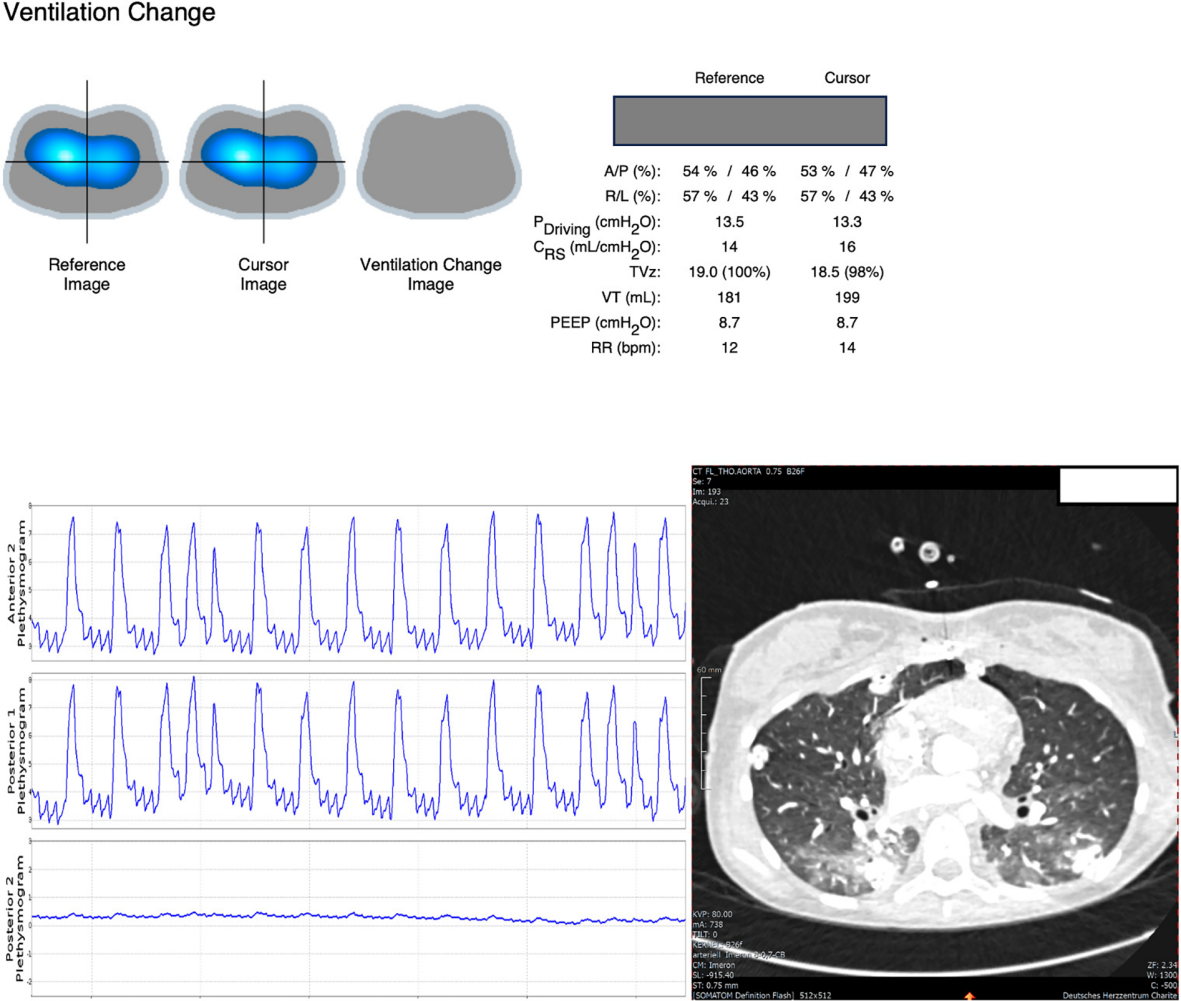
EIT signals showed no benefit of PEEP titration (neither gain nor loss in ventilation as shown in the ventilation change image on top after RM) but indicated substantial loss in posterior ventilation which is reflected by very little amplitude in the posterior plethysmogram. CT scan showed opacities in the dorsal lung.

Evaluation of regional ventilation distribution revealed reduced ventilation in the posterior lung, which was refractory to recruitment. These findings were consistent with a subsequent CT scan, which showed consolidation in the dorsal lung (Figure 2). Based on these results, we classified the patient as non-recruitable and opted for gentle ventilation (PEEP 8 cmH_2_O, driving pressure 10 cmH_2_O, RR 10 /min) and pronation (16 h/day).

The patient was successfully weaned from ECMO 3 days later, with improved lung compliance.

### Case 3

This was a 10-year-old boy with a severe bacterial infection that led to multiorgan failure, lung hemorrhage, and acute heart decompensation. Veno-arterial (VA) ECMO was implanted on-site during cardiopulmonary resuscitation, and the patient was subsequently transferred to our unit. On Day 2 of ECMO, lung compliance had decreased to <0.5 ml/cmH_2_O/kg with a FiO_2_ of 80% on ECMO.

A standardized recruitment maneuver (RM) was performed, as described previously, using a constant driving pressure of 12 cmH_2_O and a maximum PEEP of 24 cmH_2_O, followed by a stepwise return to baseline PEEP of 10 cmH_2_O. During incremental PEEP titration, EIT signals did not show increased dynamic lung strain in the dynamic images, and tidal volumes as well as lung compliance remained stable.

**Figure 3:**
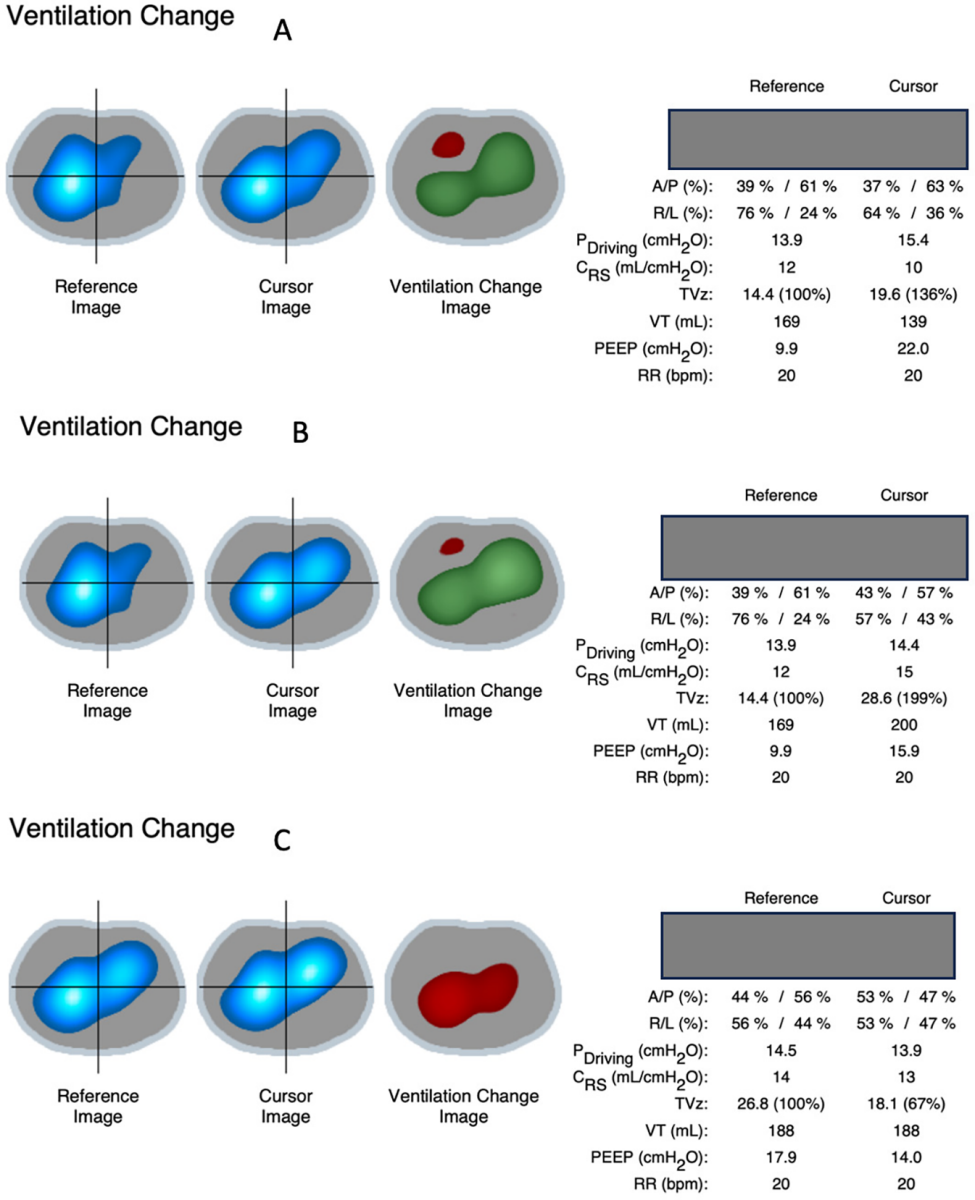
Ventilation change images based on EIT signals showed a gain in ventilation indicated by green zones (and in TVz on the right side) when increasing PEEP from 10 cmH_2_O to 22 cmH_2_O **(A)** and even more after reducing PEEP from 22 cmH_2_O to 16 cmH_2_O **(B)** decreasing PEEP from 18 to 14 cmH_2_O **(C)** resulted in decreased ventilation instead (red zone and TVz), indicating onset of de-recruitment in between. A/P, anterior/posterior; R/L, right/left; TVz, tidal volume; Pdriving, driving pressure; Crs, compliance respiratory system; TVz, tidal volume based on EIT signals; VT, tidal volume in milliliters.

During decremental PEEP titration, a clear recruitment effect was observed, with increased tidal volumes and improved compliance when comparing baseline PEEP (10 cmH_2_O) to PEEP at 22 cmH_2_O, with a more pronounced effect after reducing PEEP from 22 to 16 cmH_2_O. In contrast, decreasing PEEP from 18 to 14 cmH_2_O resulted in a decrease in tidal volumes and compliance, indicating the onset of de-recruitment (Figure 3).

After a brief re-recruitment with PEEP at 20 cmH_2_O, we decided to maintain PEEP at 16 cmH_2_O, which was considered the “best PEEP” at that time, with a constant driving pressure of 12 cmH_2_O for the following hours. Immediately after the RM, FiO_2_ and ECMO sweep gas flow were reduced based on blood gas analysis, reflecting improved ventilation as indicated by enhanced lung compliance and tidal volumes.

Despite ongoing heart failure, the pulmonary ECMO was successfully weaned 2 days later, and the patient was subsequently placed on a ventricular assist device.

### Case 4

This was a 1-month-old boy (3.8 kg) admitted to a children's hospital with tachypnea and fatigue. Following deterioration on nasal CPAP, the patient was transferred to another hospital for invasive ventilation. Intubation required multiple attempts, and the subsequent chest x-ray showed bilateral lung consolidation. The patient was then transferred to a tertiary neonatal intensive care unit, where antibiotic therapy was initiated (*Klebsiella pneumonia* was later isolated). Despite peak inspiratory pressures up to 44 cmH_2_O (PEEP 12 cmH_2_O) and 100% FiO_2_, oxygen saturation remained at 70%, and arterial pH was approximately 7.0. High doses of inotropic support (noradrenaline and adrenaline, both at 0.2 µg/kg/min) were required. Right ventricular function was preserved, and veno-venous ECMO was initiated on-site, with the patient subsequently transferred to our pediatric CICU.

Forty hours after ECMO initiation, tidal volumes were <0.5 ml/kg, and driving pressures were approximately 15 cmH_2_O. Lung recruitability was assessed using EIT during an incremental/decremental PEEP titration as previously described. Due to very low lung compliance and the absence of signs of increased dynamic lung strain on dynamic EIT images during HFOV, we decided to perform a recruitment maneuver on HFOV in the prone position. Ventilation change images could not be generated by the EIT device during conventional ventilation due to tidal volumes being <1 ml.

With a fixed amplitude of 50 cmH_2_O and a frequency of 13 Hz, we increased the mean airway pressure (Paw) by 2 cmH_2_O every 3–5 min, up to a maximum of 36 cmH_2_O, and then gradually reduced Paw to 24 cmH_2_O, which was considered optimal based on improvements in dynamic EIT images and oscillatory tidal volumes, as well as the carbon dioxide transport coefficient (DCO_2_). Even at a mean airway pressure of 36 cmH_2_O, the patient remained hemodynamically stable: arterial blood pressure slightly improved, heart rate did not increase, and central venous pressure rose only by 1 mmHg. Dynamic EIT signals did not show increased dynamic lung strain with Paw >30 cmH_2_O.

Oscillatory tidal volumes increased from <0.5 to 7 ml, and DCO_2_ improved from 20 to 60 to 700 immediately following the recruitment maneuver. Post-RM blood gas samples showed improved ventilation and gas exchange, leading to a reduction in FiO_2_ and ECMO sweep gas flow. ECMO was successfully weaned 16 h later on HFOV after the patient was repositioned supine, with Paw at 15 cmH_2_O, frequency of 14 Hz, and amplitude of 40 cmH_2_O.

**Figure 4:**
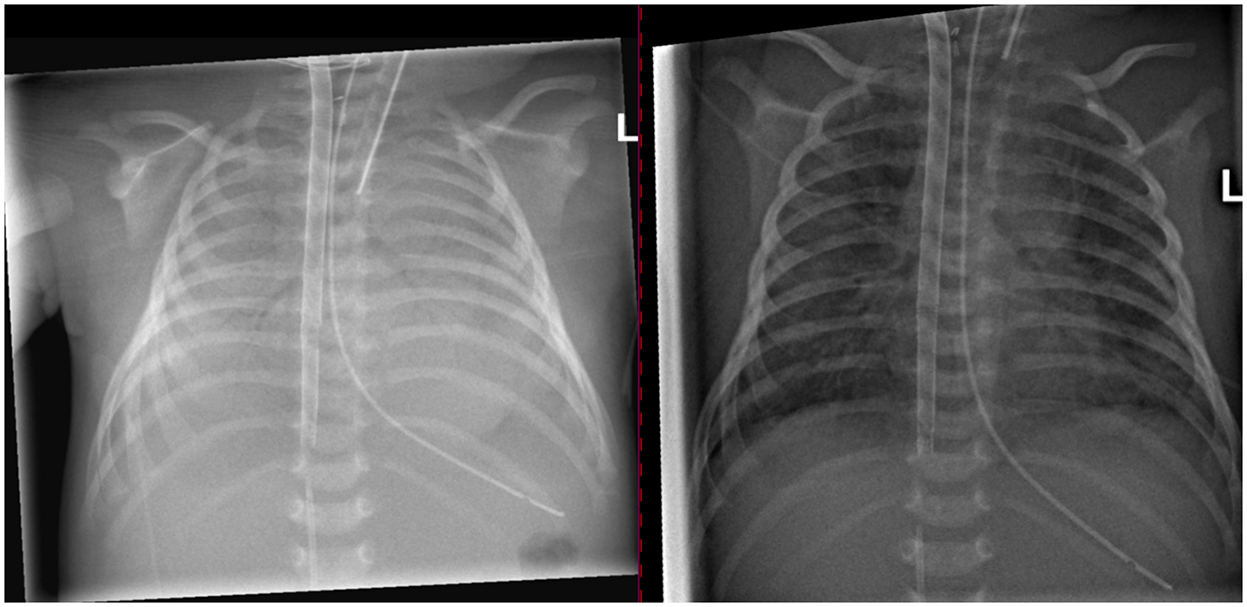
Chest x-ray prior to the recruitment maneuver on the left side and after recruitment on the right side.

The patient was transferred to the NICU the next day on conventional ventilation (PEEP of 6 cmH_2_O, pressure support of 12 cmH_2_O, and FiO_2_ of 0.3). Chest x-rays before and after the recruitment maneuver are shown in Figure 4. A short video of dynamic EIT images during HFOV at a Paw of 36 cmH_2_O is available in the online supplement.

## Discussion

This case series shows that (1) evaluation of lung recruitability with EIT is feasible. (2) In recruitable lung disease, recruitment maneuver might improve lung aeration and thereby shorten ECMO duration. (3) In non-recruitable lung disease, increases in dynamic lung strain can be detected early with EIT, thereby facilitating gentle ventilation by avoiding unnecessary lung strain.

EIT played a crucial role in monitoring dynamic lung strain during recruitment and in assessing how patients respond to the intervention (responder/non-responder).

Recently, several studies in both adult and pediatric populations have suggested that adjusting ventilation settings based on EIT findings may improve patient outcomes compared to standard treatment alone (13–15).

The recruitment maneuver in patients with recruitable lung disease led to a significant improvement in lung compliance and initiated ECMO weaning, which was successfully achieved within 48 h in Cases 3 and 4. In Case 1, lung compliance also improved markedly, but ECMO weaning was not attempted due to poor neurological prognosis. It remains unclear why no homogenization effect was observed in Case 1, despite all other parameters indicating successful recruitment. While EIT is a useful tool for assessing regional lung behavior, it is important to recognize that it may have limitations in evaluating the behavior of the entire lung, as it provides data from only a cross-sectional slice of the lung.

In Case 2, an aggressive recruitment maneuver was avoided in the context of non-recruitable lung disease, as it might have been harmful.

The advantage of EIT over conventional monitoring is the ability to continuously monitor dynamic lung strain during the maneuver, providing real-time insights into lung recruitment and potential overdistension.

Additionally, EIT was valuable in identifying the “best PEEP” levels after recruitment to prevent de-recruitment.

Our study's limitations include a small sample size and an uncontrolled design. We did not collect blood gases to obtain pCO_2_ values for each PEEP level during the recruitment maneuver, as this would have required 15 blood gas samples per patient and intervention. Additionally, we did not report individual lung compliance values for every PEEP level in each patient, as compliance remained stable during incremental PEEP titration in patients who responded positively or decreased only in Case 2, where the patient's lung was not recruitable. Instead, we chose to compare baseline settings after the recruitment maneuver to more clearly illustrate the changes following RM with identical ventilator settings. Hemodynamics did not change in any of the reported cases, individual hemodynamic values for every PEEP level have not been collected. However, given the rarity of these cases, we believe that integrating EIT into clinical practice could help personalize respiratory care at the bedside. These findings may also serve as a basis for designing larger, controlled studies on the topic.

In conclusion, EIT monitoring of lung recruitability in children with severe respiratory failure on ECMO is feasible. EIT may aid in distinguishing between recruitable and non-recruitable lung disease. Recruitment maneuvers in recruitable lung disease may shorten ECMO duration by optimizing lung aeration. Ventilation strategies that reduce ECMO and invasive mechanical ventilation duration could potentially lower the risk of impaired neurocognitive outcomes (16).

## Data Availability

The original contributions presented in the study are included in the article/Supplementary Material, further inquiries can be directed to the corresponding author.
